# Risk-stratified faecal immunochemical testing (FIT) for urgent colonoscopy in Lynch syndrome during the COVID-19 pandemic

**DOI:** 10.1093/bjsopen/zrad079

**Published:** 2023-09-05

**Authors:** Anne G Lincoln, Sally C Benton, Carolyn Piggott, Shama Riaz Sheikh, Andrew D Beggs, Leah Buckley, Bianca DeSouza, James E East, Pete Sanders, Michael Lim, Donal Sheehan, Katie Snape, Helen Hanson, John R Greenaway, John Burn, David Nylander, Menna Hawkins, Fiona Lalloo, Kate Green, Thomas J Lee, Julie Walker, Gillian Matthews, Terry Rutherford, Peter Sasieni, Kevin J Monahan

**Affiliations:** Cancer Prevention Group, School of Cancer and Pharmaceutical Sciences, King’s College London, London, UK; Department of Clinical Biochemistry and NHS Bowel Cancer Screening South of England Hub, Royal Surrey County Hospital, Berkshire and Surrey Pathology Services, Guildford, Surrey, UK; Department of Clinical Biochemistry and NHS Bowel Cancer Screening South of England Hub, Royal Surrey County Hospital, Berkshire and Surrey Pathology Services, Guildford, Surrey, UK; Cancer Prevention Group, School of Cancer and Pharmaceutical Sciences, King’s College London, London, UK; Department of Surgery, Queen Elizabeth Hospital, Birmingham, UK; Clinical Genetics, St Michael’s Hospital, Bristol, UK; Clinical Genetics, Guy’s and St Thomas’ NHS Foundation Trust, London, UK; Translational Gastroenterology Unit, John Radcliffe Hospital, Oxford, UK; Oxford NIHR Biomedical Research Centre, University of Oxford, Oxford, UK; Translational Gastroenterology Unit, John Radcliffe Hospital, Oxford, UK; Oxford NIHR Biomedical Research Centre, University of Oxford, Oxford, UK; Translational Gastroenterology Unit, John Radcliffe Hospital, Oxford, UK; Oxford NIHR Biomedical Research Centre, University of Oxford, Oxford, UK; Translational Gastroenterology Unit, John Radcliffe Hospital, Oxford, UK; Oxford NIHR Biomedical Research Centre, University of Oxford, Oxford, UK; South West Thames Regional Genetics Service, St George’s University Hospitals NHS Foundation Trust, London, UK; South West Thames Regional Genetics Service, St George’s University Hospitals NHS Foundation Trust, London, UK; Department of Gastroenterology, James Cook University Hospital, Middlesbrough, UK; Translational and Clinical Research Institute, Newcastle University, Newcastle upon Tyne, UK; Gastroenterology, Newcastle Upon Tyne Hospitals NHS Foundation Trust, Newcastle Upon Tyne, UK; Family Cancer Clinic, St Mark’s Hospital, London, UK; Manchester Centre for Genomic Medicine, Manchester University Hospitals NHS Foundation Trust, Manchester, UK; Manchester Centre for Genomic Medicine, Manchester University Hospitals NHS Foundation Trust, Manchester, UK; Gastroenterology Research, Northumbria Healthcare NHS Foundation Trust, North Shields, UK; Gastroenterology, South Tyneside and Sunderland NHS Foundation Trust, South Shields, UK; Gastroenterology, County Durham and Darlington NHS Foundation Trust, Darlington, UK; Gastroenterology, County Durham and Darlington NHS Foundation Trust, Darlington, UK; Cancer Prevention Group, School of Cancer and Pharmaceutical Sciences, King’s College London, London, UK; Family Cancer Clinic, St Mark’s Hospital, London, UK; Faculty of Medicine, Imperial College, London, UK

## Abstract

**Background:**

Lynch syndrome is a hereditary cancer disease resulting in an increased risk of colorectal cancer. Herein, findings are reported from an emergency clinical service implemented during the COVID-19 pandemic utilizing faecal immunochemical testing (‘FIT’) in Lynch syndrome patients to prioritize colonoscopy while endoscopy services were limited.

**Methods:**

An emergency service protocol was designed to improve colonoscopic surveillance access throughout the COVID-19 pandemic in England for people with Lynch syndrome when services were extremely restricted (1 March 2020 to 31 March 2021) and promoted by the English National Health Service. Requests for faecal immunochemical testing from participating centres were sent to the National Health Service Bowel Cancer Screening South of England Hub and a faecal immunochemical testing kit, faecal immunochemical testing instructions, paper-based survey, and pre-paid return envelope were sent to patients. Reports with faecal haemoglobin results were returned electronically for clinical action. Risk stratification for colonoscopy was as follows: faecal haemoglobin less than 10 µg of haemoglobin/g of faeces (µg/g)—scheduled within 6–12 weeks; and faecal haemoglobin greater than or equal to 10 µg/g—triaged via an urgent suspected cancer clinical pathway. Primary outcomes of interest included the identification of highest-risk Lynch syndrome patients and determining the impact of faecal immunochemical testing in risk-stratified colonoscopic surveillance.

**Results:**

Fifteen centres participated from June 2020 to March 2021. Uptake was 68.8 per cent amongst 558 patients invited. For 339 eligible participants analysed, 279 (82.3 per cent) had faecal haemoglobin less than 10 µg/g and 60 (17.7 per cent) had faecal haemoglobin greater than or equal to 10 µg/g. In the latter group, the diagnostic accuracy of faecal immunochemical testing was 65.9 per cent and escalation to colonoscopy was facilitated (median 49 *versus* 122 days, χ^2^ = 0.0003, *P* < 0.001).

**Conclusion:**

Faecal immunochemical testing demonstrated clinical value for Lynch syndrome patients requiring colorectal cancer surveillance during the pandemic in this descriptive report of an emergency COVID-19 response service. Further longitudinal investigation on faecal immunochemical testing efficacy in Lynch syndrome is warranted and will be examined under the ‘FIT for Lynch’ study (ISRCTN15740250).

## Teaser

This was an emergency service protocol designed to improve access to colonoscopy using faecal immunochemical testing for risk-stratified triage during the height of the COVID-19 pandemic for people with Lynch syndrome when endoscopy services were extremely limited. Requests for faecal immunochemical testing for eligible Lynch syndrome patients were made by participating National Health Service Trusts, which triggered the sending of a faecal immunochemical testing kit, faecal immunochemical testing instructions, a paper-based survey, and pre-paid return envelope from the National Health Service Bowel Cancer Screening South of England Hub in Surrey to said patients, and risk stratification for colonoscopy was as follows: faecal haemoglobin less than 10 µg/g—schedule colonoscopy within 6–12 weeks; and faecal haemoglobin greater than or equal to 10 µg/g—triage via an urgent suspected cancer clinical pathway. For 339 participants analysed, 279 (82.3 per cent) had faecal haemoglobin less than 10 µg/g and 60 (177.0 per cent) met criteria for urgent triage (faecal haemoglobin greater than or equal to 10 µg/g), with escalation to colonoscopy facilitated in the latter group of patients, with a median wait of 49 days (*versus* 122 days for f-Hb less than 10), demonstrating the clinical value of faecal immunochemical testing for patients with Lynch syndrome requiring colorectal cancer surveillance during the pandemic.

## Introduction

Lynch syndrome (LS) is an inherited cancer predisposition syndrome defined by the presence of pathogenic variants within any mismatch repair (MMR) gene (*MLH1*, *MSH2*, *MSH6*, *PMS2*) or a deletion within the *EPCAM* gene. Individuals with LS have an elevated lifetime risk of several different cancers, of which colorectal cancer (CRC) is the most common, with a cumulative lifetime risk of 10–47 per cent, dependent on the *MMR* gene variant^1^. For individuals with LS, colonoscopy may prevent or detect CRC earlier, and UK guidelines advise biennial colonoscopic surveillance beginning at age 25 or 35 years depending on genotype^2^.

During the early phase of the COVID-19 pandemic, the British Society of Gastroenterology (BSG) and the Joint Advisory Group (JAG) recommended immediate suspension of non-emergency endoscopy, including for individuals with LS^3^.

Consequently, there was a sharp decline in colonoscopy activity, with a 92 per cent overall reduction in endoscopy procedures performed in April 2020 nationally, and a 72 per cent reduction in CRC diagnoses^4^. Restoration plans in endoscopy from May 2020 focused on patients referred with suspected cancer-related symptoms and on national screening programmes^5^. Guidance for those with LS was not initially well defined before a guidance update was subsequently released in January 2021^6^.

Given the omission of LS management from early pandemic guidance^7^, a strategy recommending the emergency use of faecal immunochemical testing (FIT) for haemoglobin was proposed to identify patients with LS at the highest risk of CRC and facilitate their access to urgent colonoscopy^8^.

FIT is widely used as a quantitative non-invasive triage tool to stratify populations for further colorectal investigation in both asymptomatic patients in CRC screening programmes and symptomatic patients^9–11^. FIT had not been routinely utilized in LS patients previously and should not be considered a ‘replacement’ for normal colonoscopic surveillance. However, there is extensive evidence of a high positive predictive value (PPV) for CRC, especially at low concentrations^12–16^.

The aim of this emergency clinical service was to apply FIT as a triage tool to identify LS patients at the highest risk of prevalent CRC, and therefore escalate colonoscopic evaluation during the COVID-19 pandemic when endoscopy services were critically limited.

## Methods

### Clinical service evaluation design

This was a prospective emergency clinical service evaluation from June 2020 to March 2021 for patients with LS, which was conducted at National Health Service (NHS) England (NHSE) endoscopic and regional genetics facilities and sponsored by London North West University Healthcare NHS Trust. NHS sites were invited to use the emergency service via communication from the BSG, with a letter of support for urgent implementation provided by NHSE. Patient eligibility screening and recruitment was conducted by 15 participating NHS Trusts throughout England. The centralized mailing of FIT kits and accompanying materials, as well as the subsequent collection, analysis, result generation, and reporting, were performed by laboratory staff based at the NHS Bowel Cancer Screening South of England Hub in Guildford, Surrey (Southern Hub).

The following risk-stratified colonoscopic triage was recommended to participating centres in accordance with national COVID-19 surgical prioritization guidance^17^ to expedite colonoscopy in priority patients. These faecal haemoglobin (f-Hb) thresholds were chosen to be consistent with existing NHSE thresholds for patients with suspected CRC symptoms.

Group 1: f-Hb concentration less than 10 µg of haemoglobin/g of faeces (µg/g)—schedule patient for colonoscopy within 3 months (90 days), where local endoscopy service availability permits.
Group 2: f-Hb concentration greater than or equal to 10 µg/g—refer for triage via an urgent clinical pathway for timely colonoscopy (as per patients on a suspected cancer pathway) within 30 days.

### Participants

Eligibility for participation in this emergency service was determined by: known LS diagnosis confirmed by germline testing, irrespective of prior CRC and/or surgical history; and planned colonoscopic surveillance between 1 March 2020 and 31 March 2021. Age and genotype, which were determined in accordance with current UK LS surveillance guidelines^2^, were also considered when screening for eligibility. Patients were identified and screened for eligibility by clinical staff from the respective participating NHS Trusts by any of the following means: through an existing NHS Trust-wide LS registry; through local regional genetics service databases; or through linked endoscopy clinical records held within their local regional NHS Trusts, all of which detailed relevant genotype and age data required for screening.

Individuals who were pregnant, had prior subtotal or total colectomies, or were not fit to undergo colonoscopy were excluded from participating in this service (also see the *Supplementary Methods*).

### Faecal immunochemical testing mailings

Requests for an FIT kit (OC-SENSOR™, Eiken Chemical Co., Ltd, Tokyo, Japan) and supporting materials to be posted to eligible individuals were made by clinicians at any of the participating NHSE Trusts (also see the *Supplementary Methods* and *Appendix S1*).

### Faecal immunochemical testing analysis and disclosure of results

Returned FIT kits were promptly retrieved by Southern Hub staff for the assignment of a unique study ID and corresponding barcode label, immediately followed by the processing of collected faecal samples on the OC-SENSOR™ PLEDIA Analyser in accordance with the manufacturer’s instructions. Returned patient surveys were scanned and saved on secure network drives internal to the Southern Hub in both original un-redacted and pseudo-anonymized formats, the latter of which were printed and mailed via medical courier to the assigned project manager of this clinical service. Original paper copies were stored in a safe and secure office location within a Southern Hub office.

Sample analysis and FIT result interpretations were conducted and overseen for quality checks in accordance with the United Kingdom Accreditation Service (‘UKAS’) quality standards by registered Healthcare Scientists internal to the Southern Hub. After the verification of FIT results, individual reports were generated and electronically mailed to the requesting clinician via secure and encrypted NHS networks. Requesting clinicians in receipt of FIT reports were advised to refer patients for urgent or non-urgent colonoscopy in adherence with the predefined, risk-stratified rules.

### Outcomes of interest

The primary objectives of interest for this clinical service were to identify the highest-risk patients in this LS population and determine the impact of FIT in risk-stratified colonoscopic surveillance for this patient population. To ensure the availability and return of most or all colonoscopic and pathology (where applicable) data, analysis was predetermined to commence 1 year after the study interval end date of 31 March 2022.

Eligible patients who returned an FIT kit to the Southern Hub were classified as participants of this clinical service. Colonoscopy was performed by NHS-employed and JAG-accredited endoscopists. Bowel cleansing and colonoscopy procedures were carried out in accordance with local protocols. Colonoscopic outcomes were classified into four broad categories: no abnormalities detected (that is normal); non-advanced adenomas (NAAs); CRC; and advanced adenomas (AAs). AAs were classified in line with the latest UK post-polypectomy and post-CRC resection guidelines^18^ and defined as adenomas greater than or equal to 10 mm and/or with high-grade dysplasia. In the present analysis, adenomas having tubulovillous or villous histology were classified as AAs. Advanced colorectal neoplasia (ACN) describes either AAs or CRC. The adenoma detection rate (ADR) (NAAs and AAs) and AA detection rate (AADR; AAs only) were calculated. Colonoscopic yield was evaluated at different f-Hb thresholds. The limit of quantification of f-Hb was 6 µg/g^19^.

Additionally, relevant clinical data were also collected at the time of colonoscopy, including presence of anastomosis and gastrointestinal co-morbidities.

### Statistical analysis

Descriptive and analytic statistics were carried out utilizing Stata/SE v. 17.0 (StataCorp LP, College Station, TX, USA). Numbers and percentages for the baseline characteristics of participants (sex, age (calculated at time of FIT kit request), and the time from FIT kit dispatch to date of colonoscopy) were calculated for the outcomes (normal, NAAs, AAs, and CRC) overall and for patients with f-Hb greater than or equal to 10 µg/g.

Logistic regression analysis was used to compute estimates and symmetric confidence limits of sensitivity, specificity, PPV, and negative predictive value (NPV). The log OR from the logistic regression was converted to a proportion by taking the inverse of those values, which was then multiplied by 100 to arrive at the percentage.

Group statistics and an independent samples *t* test (*P* ≤ 0.050) were generated to calculate the statistical significance of age between those who returned an FIT kit and those who did not. The risk ratio of a CRC diagnosis with f-Hb greater than or equal to 10 µg/g was calculated using a two-tailed Fisher’s exact test at a significance level of 5 per cent.

A receiver operating characteristic (ROC) curve was produced using the ‘*senspec*’ command^20^ in Stata to calculate true positive (TP) and false positive (FP) rates, which were then plotted using a scatter graph. The area under the curve (AUC) and 95 per cent confidence intervals (c.i.) were calculated using the ‘*somersd*’ command in Stata^21^.

Further, differences in time to colonoscopy from the date FIT kits were dispatched from the Southern Hub at cut-offs of f-Hb less than 10 µg/g *versus* f-Hb greater than or equal to 10 µg/g and at cut-offs of f-Hb less than 6 µg/g *versus* f-Hb 6 to less than 10 µg/g *versus* f-Hb greater than or equal to 10 µg/g were analysed (secondary analysis) by means of the Kaplan–Meier method and log rank test. Time was defined as the time from the date FIT kits were dispatched from the Southern Hub to colonoscopy (days) up to 120 days. The log rank test (at a significance level of 5 per cent) was used to test the null hypothesis that the proportions in the different groups were identical.

Pearson’s chi-squared test was used to test for an association between FIT results and the presence of a surgical anastomosis, with significance pre-specified at <0.050.

## Results

### Characteristics of clinical service participants

**Fig. 1:**
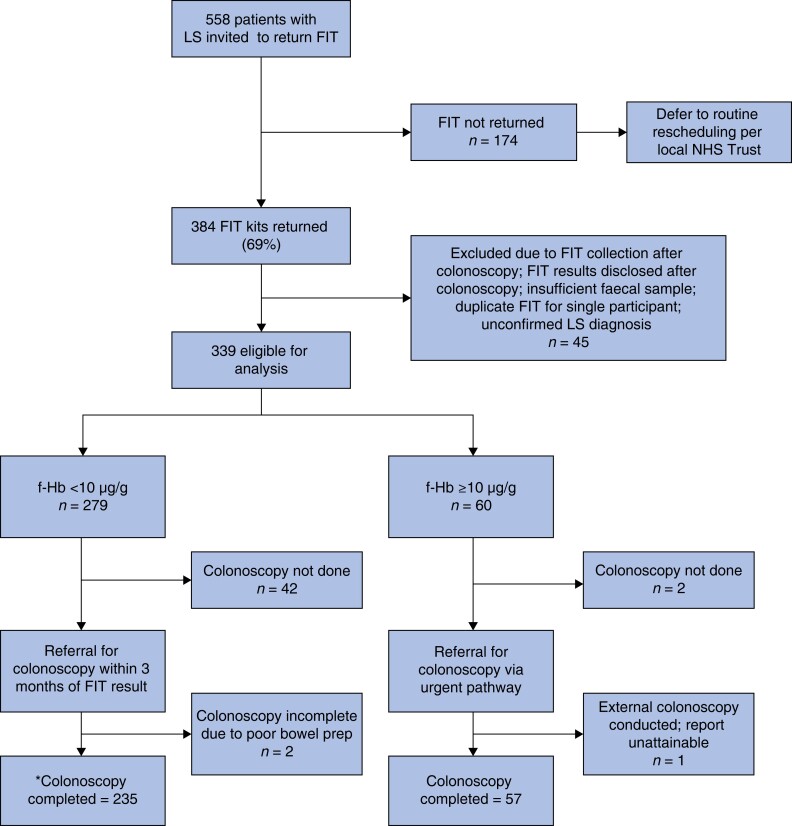
Flow chart depicting risk-stratified colonoscopy referral design using faecal haemoglobin of greater than or equal to 10 µg/g to indicate a positive test in a cohort of Lynch syndrome patients who returned a faecal immunochemical testing kit as part of this emergency clinical service *Of the 235 participants triaged via Group 1 (f-Hb less than 10 µg/g), 2 were referred for and completed flexi-sigmoidoscopy and 1 had a CT colonography likely due to resource limitations and/or participant tolerance. LS, Lynch syndrome; FIT, faecal immunochemical testing; NHS, National Health Service; f-Hb, faecal haemoglobin.

Between 9 June 2020 and 31 March 2021, 558 LS individuals were invited across 15 participating NHSE Trusts (*Fig. 1*), after initial identification via internal registries, local regional genetics service databases, or through linked endoscopy clinical records internal to their local regional NHS Trusts, and subsequently screened for eligibility. An uptake of 68.8 per cent (384 of 558) was observed. The mean(s.d.) age of this cohort at the time their FIT kit was dispatched from the Southern Hub was 51.6(14.1) years, of whom women accounted for the majority (55.0 per cent), though sex was unknown or not reported for 66 (12.0 per cent) patients (*Table S1*). Individuals with a pathogenic variant in *MLH1*, *MSH2*, *MSH6*, *PMS2*, and *EPCAM* comprised 34.0, 35.0, 19.0, 7.0, and 4.0 per cent of the participants respectively. For 22 (4.0 per cent) patients, LS genotype data were not available, and these patients were removed from the final analysis.

### Faecal immunochemical testing results

Sixty (17.7 per cent) participants had f-Hb greater than or equal to 10 µg/g and met criteria for urgent colonoscopic triage per protocol. FIT positivity increased from 17.7 per cent at a cut-off of greater than or equal to 10 µg/g to 23.9 per cent at a cut-off of greater than or equal to 6 µg/g (including 6 to less than 10 µg/g and greater than or equal to 10 µg/g positivity rates; *Table 1*). Out of 384 patients who returned an FIT kit, 339 individuals met the eligibility criteria (*Supplementary Results* and *Table S2*). Patients returning FIT kits were older (mean(s.d.) = 51(13.99) years, *P* < 0.001; two-sample *t* test with unequal variances) compared with those who did not return their FIT kits (mean(s.d.) = 45(12.81) years). (*Supplementary Results* and *Table S3*).

**Table 1:** Colonoscopic yield by faecal immunochemical testing thresholds of less than 6 µg/g, 6 to less than 10 µg/g, and greater than or equal to 10 µg/g

FIT threshold (µg/g)	Total	Overall instances of participants with adenomas*	AAs	CRC	NAD	ADR, %	AN-DR, %	Pending colonoscopy or missing data, *n*
<6	258 (76.1)	99 (38.4)	19 (7.4)	3 (1.2)	115 (44.6)	45.6	8.8	41
6 to <10	21 (6.2)	3 (14.3)	0 (0.0)	0 (0.0)	15 (71.4)	16.7	0.0	3
≥10	60 (17.7)	28 (46.7)	8 (13.3)	4 (6.7)	25 (41.7)	49.1	14.0	3
Total	339	130 (44.5)	27 (9.2)	7 (2.4)	155 (53.1)	44.5	9.2	47

*Includes non-advanced adenomas as well as advanced adenomas (AAs). Values are *n* (%) unless otherwise indicated. In the ‘total’ column, listed percentages are of the overall total (339), whereas all other percentages are reflective of row total. Please note that ‘overall cases with adenomas’ includes AAs, but not CRC. FIT, faecal immunochemical testing; CRC, colorectal cancer; NAD, no abnormalities detected; ADR, adenoma detection rate; AN-DR, advanced neoplasia detection rate.

### Colonoscopic outcomes

At the time of analysis in March 2022, colonoscopic data were provided for 87.0 per cent (295 of 339) of clinical service participants (*Table 1*). Three patients were excluded from analysis where the report was unattainable, or the procedure was incomplete. The ADR and advanced neoplasia detection rate (AN-DR) for all participants were 46.9 and 9.2 per cent respectively. The presence of surgical anastomosis was found to be non-significantly associated with f-Hb greater than or equal to 10 µg/g (17 of 71 (23.9 per cent) when compared with f-Hb less than 10 µg/g (32 of 225 (14.2 per cent), χ^2^ = 3.69, *P* = 0.055). In 279 participants (or 279 colonoscopies) with f-Hb less than 10 µg/g, 102 adenomas (ADR = 43.4 per cent), 19 AAs (AN-DR = 8.1 per cent), and 3 CRCs were detected. For those with f-Hb greater than or equal to 10 µg/g, 28 adenomas (ADR = 49.1 per cent), 8 AAs (AN-DR = 14.0 per cent), and 4 CRCs were identified. The risk ratio for having a CRC with f-Hb greater than or equal to 10 µg/g was 6.2 per cent (95 per cent c.i. 1.4 to 27.0 per cent) (*P* = 0.021).

### Diagnostic accuracy of faecal immunochemical testing

**Fig. 2:**
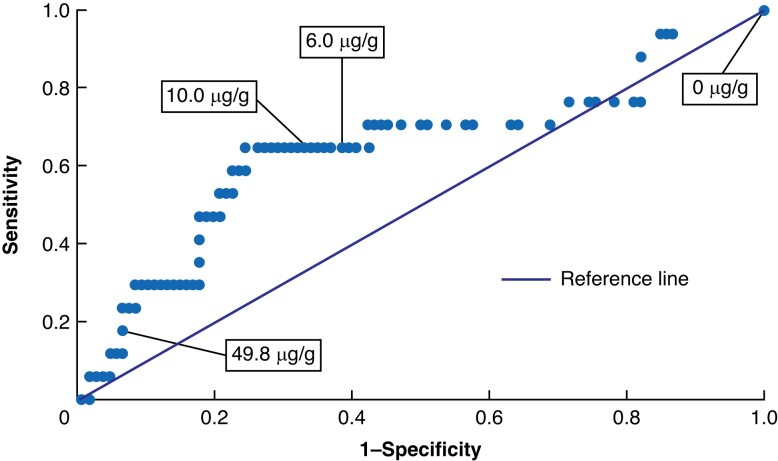
Receiver operating characteristic (ROC) curve depicting the performance of faecal immunochemical testing at various faecal immunochemical testing thresholds Area under the curve = 0.66 (95% c.i. 0.53,0.79).

The diagnostic accuracy of FIT for those who completed a colonoscopy within the advised time frame was analysed using an ROC curve (*Fig. 2*). For the detection of ACN, the AUC was 0.7 (95 per cent c.i. 0.5 to 0.8). *Table 2* describes the diagnostic accuracy of FIT in the detection of ACN within 90 days, categorized by FIT thresholds of greater than or equal to 6 µg/g and greater than or equal to 10 µg/g. FIT accuracy was determined to be 61.0 and 65.9 per cent for greater than or equal to 6 µg/g and greater than or equal to 10 µg/g respectively. For both greater than or equal to 6 µg/g and greater than or equal to 10 µg/g, sensitivity was 64.7 per cent (95 per cent c.i. 38.3 to 85.8 per cent). Specificity was 66.0 per cent (95 per cent c.i. 56.2 to 75.0 per cent) for greater than or equal to 10 µg/g and 60.4 per cent (95 per cent c.i. 50.4 to 69.8 per cent) for greater than or equal to 6 µg/g. PPV and NPV improved by 2.6 and 0.7 percentage points respectively in greater than or equal to 10 µg/g *versus* greater than or equal to 6 µg/g.

**Table 2:** Diagnostic accuracy of faecal immunochemical testing for the detection of advanced colorectal neoplasia within 90 days by greater than or equal to 6 µg/g and greater than or equal to 10 µg/g thresholds

FIT threshold (µg/g)	Accuracy, %	Sensitivity	Specificity	PPV	NPV	TP, *n*	FN, *n*	FP, *n*	TN, *n*
≥6	61.0	64.7 (38.3,85.8)	60.4 (50.4,69.8)	20.8 (14.7,28.6)	91.4 (84.6,95.4)	11	6	42	64
≥10	65.9	64.7 (38.3,85.8)	66.0 (56.2,75.0)	23.4 (16.4,32.2)	92.1 (85.8,95.8)	11	6	36	70

Values are % (95% c.i.) unless otherwise indicated. FIT, faecal immunochemical testing; PPV, predictive value; NPV, negative predictive value; FP, false positive; TP, true positive; TN, true negative; FN, false negative.

### Colonoscopic waiting time by faecal immunochemical testing results

The mean(s.d.) time to colonoscopy for all participants who returned an FIT kit and subsequently underwent colonoscopy (292 participants) was 153(116.65) days, with a median of 122 days (*Table 3*). Participants with f-Hb greater than or equal to 10 µg/g who were triaged to urgent colonoscopy had a mean(s.d.) wait of 77(85.0) days, with a median of 49 days, with 12 (20.0 per cent) having a colonoscopy performed within the recommended time frame of 30 days. In those with f-Hb less than 10 µg/g, the mean(s.d.) waiting time was 153(116.6) days, with a median of 122 days, though three such participants who were later found to have CRC were referred for colonoscopy outside the protocol time frame of within 90 days with a mean(s.d.) of 294(74) days. Overall, the majority (58.3 per cent) of those with f-Hb less than 10 µg/g were not referred for colonoscopy within the service’s recommended time frame. In total, 44 patients with f-Hb less than 10 µg/g deferred their colonoscopy (beyond study follow-up) in comparison with 2 colonoscopy deferrals for those with f-Hb greater than or equal to 10 µg/g (*P* = 0.010).

**Table 3:** Time (days) to colonoscopy for faecal haemoglobin less than 10 µg/g and for faecal haemoglobin greater than or equal to 10 µg/g by colonoscopic outcome

Days	Overall	f-Hb <10 µg/g				f-Hb ≥10 µg/g			
		Normal	NAAs	AAs	CRC	Normal	NAAs	AAs	CRC
0–30	86 (29.5)	26 (20.0)	19 (22.9)	5 (26.3)	0 (0.0)	16 (64.0)	13 (65.0)	4 (50.0)	3 (75.0)
31–60	58 (19.9)	21 (16.2)	24 (29.0)	1 (5.3)	0 (0.0)	4 (16.0)	4 (20.0)	3 (37.5)	1 (25.0)
61–90	2 (0.7)	1 (0.8)	0 (0.0)	1 (5.3)	0 (0.0)	0 (0.0)	0 (0.0)	0 (0.0)	0 (0.0)
>90	146 (50)	82 (63.1)	40 (48.2)	12 (63.2)	3 (100.0)	5 (20.0)	3 (15.0)	1 (12.5)	0 (0.0)
Total, *n*	292	130	83	19	3	25	20	8	4

Values are *n* (%) unless otherwise indicated. f-Hb, faecal haemoglobin; NAAs, non-advanced adenomas; AAs, advanced adenomas; CRC, colorectal cancer.

### Kaplan–Meier analysis of time to colonoscopy

**Fig. 3:**
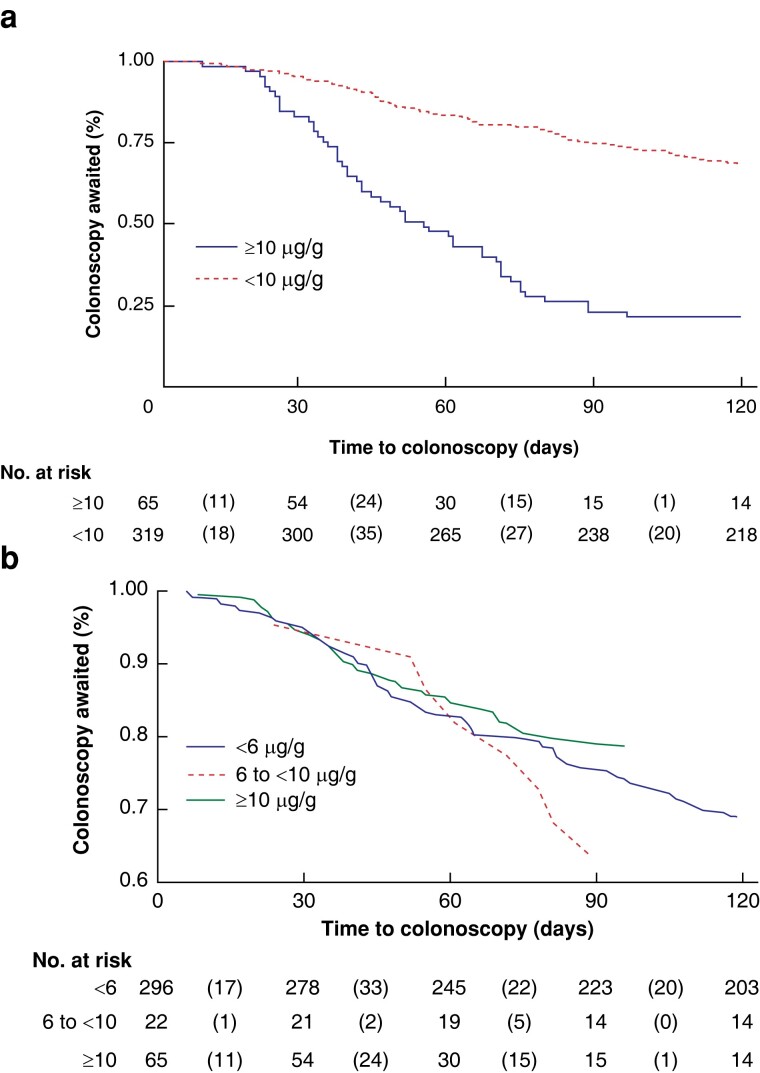
Kaplan–Meier curves **a** Line graph with survivals calculated by utilising the Kaplan–Meier (KM) method to examine the proportion of participants by FIT result (f-Hb less than 10 µg/g *versus* f-Hb greater than or equal to 10 µg/g) who completed colonoscopy within 120 days from time of FIT dispatch. **b** Line graph with survivals calculated by utilising the KM method to examine the proportion of participants who completed colonoscopy within 120 days from time of FIT dispatch by the following FIT thresholds: f-Hb less than 6 µg/g; f-Hb 6 to less than 10 µg/g; and f-Hb greater than or equal to 10 µg/g. FIT, faecal immunochemical testing; f-Hb, faecal haemoglobin.

Time to colonoscopy (days) from date of FIT dispatch was further assessed by utilising the Kaplan–Meier (KM) method on participants with f-Hb greater than or equal to 10 µg/g *versus* participants with f-Hb less than 10 µg/g (*Fig. 3a*) to examine the proportion of participants from the respective groups completing colonoscopy within 120 days, approximately 1 month outside of the recommended time frame. Within the f-Hb greater than or equal to 10 µg/g group, the proportion of participants who completed a colonoscopy just beyond the specified 90-day/3-month time frame was significantly higher than within the f-Hb less than 10 µg/g group (66.4 *versus* 33.1 per cent, χ^2^ = 13.72 and *P* < 0.001). Kaplan–Meier analysis was also used to examine the same outcome between the FIT result classifications of less than 6 µg/g, 6 to less than 10 µg/g, and greater than or equal to 10 µg/g (*Fig. 3b*). In this instance, a significantly higher proportion of participants was also referred for and completed colonoscopy in a timely manner for the f-Hb greater than or equal to 10 µg/g group compared with the other two groups (less than 6 µg/g and 6 to less than 10 µg/g) (χ^2^ = 13.88, *P* < 0.001).

## Discussion

Despite the severely limited capacity of endoscopy services during the COVID-19 pandemic in the UK^3,22,23^, these findings suggest that FIT demonstrated clinical value as a risk-stratified method in this emergency service. FIT informed timely colonoscopic triage for those with f-Hb results of greater than or equal to 10 µg/g and significantly expedited the time to colonoscopy completion compared with those with f-Hb results of less than 10 µg/g.

Given the intended use of FIT to inform and augment timely colonoscopy as a ‘point-in-time’ test, the diagnostic accuracy of FIT in these participants was analysed for those who completed a colonoscopy within the recommended 90-day window, which yielded sensitivity, specificity, PPV, and NPV estimates of 65, 66, 23, and 92 per cent respectively.

As such, the three participants with f-Hb results of less than 10 µg/g who were later diagnosed with CRC did not undergo colonoscopy per protocol given the waiting time that exceeded the recommended 90 days with a mean(s.d.) of 294(74) days. Moreover, these cancers were small (less than 15 mm in dimension) and, given the long interval to diagnosis from FIT, could well be considered *de novo*, as opposed to ‘FIT-negative’ CRC. However, a non-invasive tool such as FIT may be misinterpreted by patients or clinicians, and this risk needs to be incorporated into the future design of the use of FIT as an adjunct to colonoscopy.

Nevertheless, as a real-world clinical service designed and deployed to address the urgent need for ongoing CRC surveillance in a sizeable LS population, potential advantages were observed. The collection of real-world surveillance colonoscopy and early diagnostic accuracy data during the global pandemic in these high-risk participants enabled important clinical insights, and findings that may inform future work.

Variable access for LS patients to colonoscopy across participating NHS Trusts throughout the duration of this emergency clinical service was noted. In response to national initiatives to restore endoscopy services to pre-COVID-19 levels beginning in the latter half of 2020^24^, individual endoscopy services re-established standard surveillance and screening programmes whilst minimizing colonoscopy waiting lists at variable rates. This may have resulted in variability in colonoscopy waiting times for a subset of clinical service LS participants, thus slightly skewing colonoscopy triaging timelines in favour of NHS Trusts who may have had a wider capacity and access to additional endoscopic resources during this time.

Limitations of this project are recognized within this pragmatic project report. First, validated colonoscopy outcome data were missing for a small subset of participants. Of the 339 participants, 47 were missing colonoscopy outcome data at the time of data analysis, as participants were still awaiting their colonoscopy, or patients or clinicians decided to forgo colonoscopy altogether. Key insights to better understand barriers may be gleaned from the participant paper-based surveys that adopted a validated framework^25^ to summarize the attitude and perceptions of FIT of these participants and will be evaluated separately.

Second, the reasons for which non-participants chose to forgo participation in this service by opting out of using the provided FIT kit were not accessible and available for analysis. Several scenarios may have accounted for decisions to forgo participation, which may include general misinterpretations surrounding the clinical service, forgetting to provide and return a sample, already having an upcoming colonoscopy appointment, or, for older or more vulnerable populations, a concern in leaving their home during ongoing lockdown restrictions.

Further, any information pertaining to expected surveillance colonoscopy dates before the onset of pandemic restrictions could not be verified, albeit indicative of the ‘real-world’ design of this emergency clinical service.

Finally, most participating genetics-based services were not aware when and where their referred patients were completing their colonoscopy. Coordination between genetics and endoscopy services was not observed at most participating centres, with only a minority maintaining a comprehensive LS patient registry, the absence of which may have had an impact on patient outcomes, including CRC incidence and mortality^26^. The low response rate from eligible clinical services and evidence of the absence of a coordinated national LS registry and networked services throughout NHSE are being addressed via the Genomic Medical Service Alliance (‘GMSA’) national LS project^27^.

Though several studies have explored the augmentative potential of FIT for surveillance colonoscopies in individuals at elevated risk of CRC, further longitudinal research in LS patient populations is warranted to evaluate the diagnostic accuracy of FIT in LS. Specifically, future studies should consider lower FIT thresholds for this patient population given the suboptimal AUC for 10 µg/g, as depicted in *Fig. 2*. Moreover, subsequent studies should explore whether existing LS surveillance guidelines may be adapted to include FIT as an augmentative device to colonoscopy, which is presently being explored through the multi-institutional prospective feasibility study known as ‘FIT for Lynch’^28^.

Additional key questions remain, which should also be explored through future research, such as ‘do LS CRCs bleed?’ or whether they may potentially evade detection using FIT due to biological factors, though it should be noted that microvessels in LS adenomas are similar to those in non-LS adenomas^29^. On the other hand, FIT may be used to personalize surveillance and follow-up while other non-invasive methodology may augment colonoscopy, for example the microbiome or other biomarkers, potentially improving the efficacy of FIT in individuals with LS. The findings that suggest an association between surgical anastomosis and a ‘positive’ FIT result with an f-Hb threshold of greater than or equal to 10 µg/g should be considered when designing future studies. Also, the recommended use of aspirin in individuals with LS^2^ may potentially influence FIT results in these participants and should be explored in future studies.

Ultimately, the integration of such questions into future research study designs will facilitate a deeper understanding of the potential role of FIT as a non-invasive modality in augmenting colonoscopy as the gold standard in CRC surveillance.

## Supplementary Material

zrad079_Supplementary_Data

## Data Availability

The data sets generated during and/or analysed during this study may be available from the corresponding author on reasonable request.
