# Erratum to: Risk-stratified faecal immunochemical testing (FIT) for urgent colonoscopy in Lynch syndrome during the COVID-19 pandemic

**DOI:** 10.1093/bjsopen/zrad153

**Published:** 2023-11-29

This is an erratum to: Anne G Lincoln, Sally C Benton, Carolyn Piggott, Shama Riaz Sheikh, Andrew D Beggs, Leah Buckley, Bianca DeSouza, James E East, Pete Sanders, Michael Lim, Donal Sheehan, Katie Snape, Helen Hanson, John R Greenaway, John Burn, David Nylander, Menna Hawkins, Fiona Lalloo, Kate Green, Thomas J Lee, Julie Walker, Gillian Matthews, Terry Rutherford, Peter Sasieni, Kevin J Monahan, Risk-stratified faecal immunochemical testing (FIT) for urgent colonoscopy in Lynch syndrome during the COVID-19 pandemic, *BJS Open*, Volume 7, Issue 5, October 2023, zrad079,

In the originally published version of this manuscript, there were errors in section a of Figure 3a/b.

Figure 3a should read:

**Figure:**
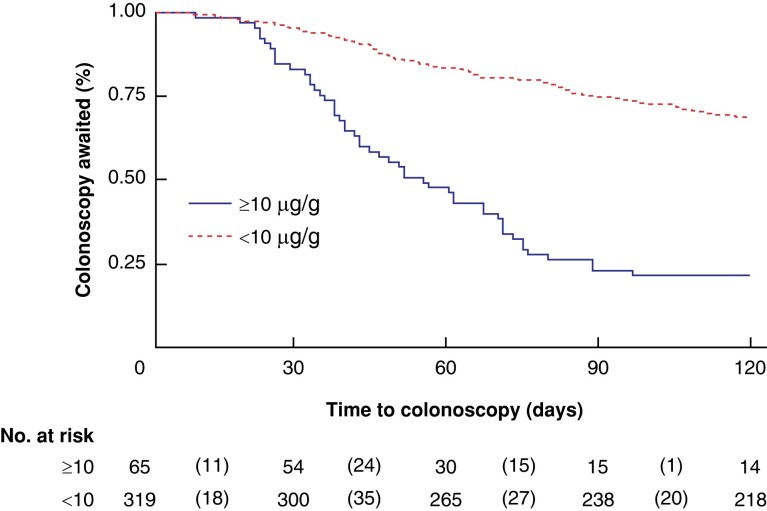


instead of:

**Figure:**
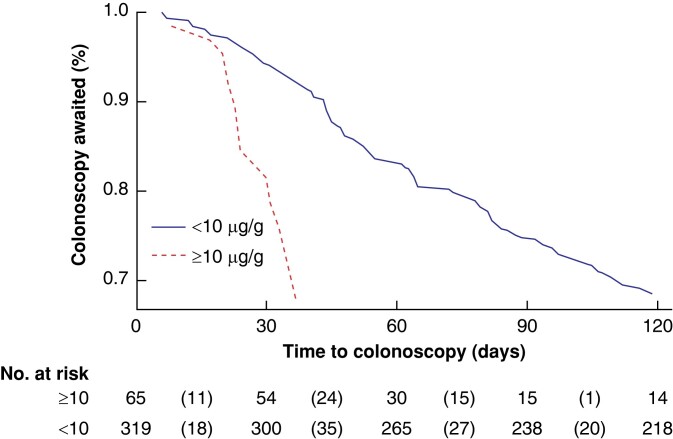


The errors have been emended in the article.

