# Supplementary material for: Risk-stratified faecal immunochemical testing (FIT) for urgent colonoscopy in Lynch syndrome during the COVID-19 pandemic
Source: BJS Open. 2023 Sep 5;7(5):zrad079. doi: 10.1093/bjsopen/zrad079 (PMC10478750; doi:10.1093/bjsopen/zrad079)
Supplement: zrad079_Supplementary_Data [file zrad079_supplementary_data.docx]

**Risk-stratified faecal immunochemical testing (FIT) for urgent colonoscopy in Lynch Syndrome during the COVID-19 pandemic**

Anne G Lincoln^1^, Sally C Benton^2^, Carolyn Piggott^2^, Shama Sheikh^1^, Andrew Beggs^3^, Leah Buckley^4^, Bianca DeSouza^5^, James E East^6,7^, Pete Sanders^6,7^, Michael Lim^6,7^, Katie Snape^8^, Helen Hanson^8^, John R Greenaway^9^, John Burn^10^, David Nylander^11^, Fiona Lalloo^12^, Kate Green^12^, Thomas J Lee^13^, Julie Walker^14^, Gillian Matthews^15^, Terry Rutherford^15^, Peter Sasieni^1^, Kevin J Monahan*^16,17^

^1^ Cancer Prevention Group, School of Cancer and Pharmaceutical Sciences, King's College London, London, UK

^2^ Department of Clinical Biochemistry and NHS Bowel Cancer Screening South of England Hub, Royal Surrey County Hospital, Berkshire and Surrey Pathology Services, Guildford, Surrey, UK

^3^ Department of Surgery, Queen Elizabeth Hospital, Birmingham, UK

^4^ Clinical Genetics, St Michael’s Hospital, Bristol, UK

^5^ Clinical Genetics, Guy's and St Thomas' NHS Foundation Trust, London, UK

^6^ Translational Gastroenterology Unit, John Radcliffe Hospital, Oxford, UK

^7^ Oxford NIHR Biomedical Research Centre, University of Oxford, Oxford, UK

^8^ South West Thames Regional Genetics Service, St George's University Hospitals NHS Foundation Trust, London, UK

^9^ Department of Gastroenterology, James Cook University Hospital, Middlesbrough, UK

^10^ Translational and Clinical Research Institute, Newcastle University, Newcastle upon Tyne, UK

^11^ Gastroenterology, Newcastle Upon Tyne Hospitals NHS Foundation Trust, Newcastle Upon Tyne, UK

^12^ Manchester Centre for Genomic Medicine, Manchester University Hospitals NHS Foundation Trust, Manchester, UK

^13^ Gastroenterology Research, Northumbria Healthcare NHS Foundation Trust, North Shields, UK

^14^ Gastroenterology, South Tyneside and Sunderland NHS Foundation Trust, South Shields, UK

^15^ Gastroenterology, County Durham and Darlington NHS Foundation Trust, Darlington, UK

^16^ Family Cancer Clinic, St Mark's Hospital, London, UK

^17^ Faculty of Medicine, Imperial College, London, UK

***Corresponding author:** Kevin J Monahan [k.monahan@imperial.ac.uk](mailto:k.monahan@imperial.ac.uk); **ORCID ID:** 0000-0002-7918-4003; **Twitter:** kevinjmonahan

**Supplementary Materials – Index**

| **Supplementary Methods** |  |
| --- | --- |
| 1. A detailed summary of the processes and materials which were mailed to eligible LS patients via the Southern Hub. 2. An expanded summary of eligibility criteria, including exclusion criteria detailing cases removed from final statistical analysis   **Supplementary Results**   1. Statistical significance of variance in age between those who returned a FIT kit and those who did not. | *pg 2-3*  *pg 3*  *pg 3* |
| **Supplementary Appendixes** |  |
| 1. **Appendix S1:** A paper-based survey comprised of two validated questionnaires designed to assess attitudes of acceptability^(1)^ towards FIT in this setting, in addition to open-ended questions allowing for additional feedback. | *pg 3-5* |
| **Supplementary Figures and Tables** |  |
| 1. **Table S1:** Summary of findings from a two-sample t-test with unequal variances (*p*-value ≤0.05) to examine variance of age between those who returned a FIT kit and those who did not. 2. **Table S2:** Demographic and clinical characteristics of those who returned a FIT kit. 3. **Table S3:** Demographic characteristics and colonoscopic outcomes for participants   (n=339) by overall FIT results and those with f-Hb above threshold (≥10µg/g).   1. **References** | *pg 6*    *pg 6-7*  *pg 7*  *pg 8* |

**Supplementary Methods**

1. Upon initial identification of any eligible patients, participating NHS Trusts were requested to complete a Microsoft® Excel template containing relevant patient details to include 1) the patient’s unique NHS number 2) Patient Hospital Number 3) Date of Birth (DOB) 4) Patient Forename 5) Patient Surname 6) Lynch Syndrome genotype 7) Patient Mailing Address 8) Patient Post Code 9) relevant service clinician (requestor) details, and 10) the date of the request for a FIT mailing.

Populated templates were then electronically mailed via secure, encrypted NHS pathways from the respective NHS Trusts to the laboratory team based at the NHS Bowel Cancer Screening South of England Hub (Southern Hub), Berkshire and Surrey Pathology Services, which acted as the central laboratory for this clinical service. Upon receipt of completed templates, the laboratory posted a FIT pack to individual patients. The FIT packs were comprised of: a) A single FIT Kit (OC-Sensor™, Eiken Chemical Co. Ltd, Tokyo, Japan) with an affixed label containing the recipients’ name, NHS number, and DOB b) a personalised test request form from the patient’s clinician c) a patient information leaflet d) Instructions for patients e) a double-sided, paper-based survey (Appendix S1) which was comprised of several validated questionnaires designed to assess attitudes of acceptability^(1)^ towards FIT in this setting, as well as open-ended questions allowing for any additional feedback f) a biohazard bag for FIT collection, and g) a pre-paid return envelope for the return of completed FIT kits and all accompanying materials to be sent back to the Southern Hub.

A record of mailing recipients was logged by Southern Hub staff within an internal and secure electronic database for tracking purposes.

1. Individuals with a known diagnosis of LS who were due for a standard of care (SOC) surveillance colonoscopy at any NHSE facility between 1 March 2020 – 31^st^ March 2021, and were competent in English were eligible for this service.

Individuals were excluded from final statistical analysis if they met any of the following criteria: i. faecal sample collected after colonoscopy; ii. faecal sample was determined to be insufficient for processing; iii. duplicate FIT from a single participant or; iv. individuals were found to not have a confirmed diagnosis of LS by either having a variant of unknown significance (VUS), solely having somatic *MMR* mutations on tumour tissue (vs. germline), or solely having a family history which was suggestive of LS.

**Supplementary Results**

1. Variance in age was examined between those who returned a FIT kit (n=339) and those who did not (n=174) (Table 2). The average age of those who returned a FIT kit was approximately 51 (SD = 13.99; Std. Error Mean = 0.76), whereas the average age of those who did not return a FIT kit appeared to be slightly younger at approximately 45 years (SD = 12.81; Std. Error Mean = 0.97). Statistical significance between both groups (p-value ≤0.05) was identified through a two-sample t test with unequal variances.

**Supplementary Appendixes**

**Appendix S1**

**Title:** Paper-based survey to assess attitudes and acceptability of FIT in individuals with LS.

Full Name: ________________ DOB: __\__\_____

**Patient Questionnaire:**

**“*Rapid evaluation of Faecal immunochemical testing (FIT) levels in individuals with a Lynch Syndrome pathogenic variant to determine a revised threshold for colonoscopy in response to the COVID-19 pandemic*”**

|  | **Please indicate how much you agree with each of the statements below** | | | | | | | |
| --- | --- | --- | --- | --- | --- | --- | --- | --- |
|  | | **Strongly disagree** | **Disagree** | **Indifferent; Neither Agree nor Disagree** | **Agree** | | **Strongly agree** | **N/A** |
| I have been anxious about my routine colonoscopy being cancelled or postponed due to COVID-19. | | □ | □ | □ | | □ | □ | □ |
| Having the option of using the FIT kit is reassuring. | | □ | □ | □ | | □ | □ | □ |
| I like the idea of doing the kit in the comfort of my own home | | □ | □ | □ | | □ | □ | □ |
| The NHS makes me feel cared for by offering me this FIT kit while I wait for a colonoscopy. | | □ | □ | □ | | □ | □ | □ |
| I trust that the result of the FIT test can be used to decide when I need a colonoscopy. | | □ | □ | □ | | □ | □ | □ |
| Doing a FIT kit every year, as well as having a colonoscopy every two years, would improve my Lynch Syndrome surveillance. | | □ | □ | □ | | □ | □ | □ |

**Supplementary Figures and Tables**

**Table S1.**

**Title:** Reported LS genotypes of individuals who met initial eligibility for this emergency clinical service categorised by sex and age

|  | **Overall, *n* (%)** | **LS Genotype** | | | | | | |
| --- | --- | --- | --- | --- | --- | --- | --- | --- |
| **Characteristics** | (n (total kits sent)) = **558** | MLH1 | MLH1 & PMS2** | MSH2 | MSH6 | PMS2 | EPCAM /  EPCAM & MSH2 | Unknown  (*Family History (FH) suggestive of LS*)) |
| **Sex** |  |  |  |  |  |  |  |  |
| Male | 188 (33.7) | 61 | 0 | 55 | 41 | 20 | 1 | 10 |
| Female | 304 (54.5) | 101 | 1 | 115 | 59 | 19 | 1 | 7 |
| Unknown/  Unreported | 66 (11.9) | 25 | 2 | 25 | 7 | 2 | 0 | 5 |
| **Age Range** |  |  |  |  |  |  |  |  |
| ≤25 | 12 (2.2) | 1 | 0 | 10 | 0 | 1 | 0 | 0 |
| 26-35 | 98 (17.6) | 36 | 0 | 40 | 15 | 4 | 1 | 2 |
| 36-45 | 127 (22.8) | 48 | 1 | 41 | 22 | 9 | 0 | 6 |
| 46-55 | 132 (23.7) | 47 | 0 | 47 | 23 | 8 | 1 | 6 |
| 56-65 | 99 (17.7) | 27 | 2 | 31 | 25 | 9 | 1 | 4 |
| 66-75 | 77 (13.8) | 24 | 0 | 20 | 21 | 9 | 0 | 3 |
| >75 | 13 (2.3) | 4 | 0 | 6 | 1 | 1 | 0 | 1 |

All numbers are expressed as values and (%).

**Individuals noted as having *MLH1 & PMS2* variants on request templates sent to the Southern Hub were later determined to be “Lynch-like” based on somatic reports alone and ultimately excluded from final analysis for not meeting the specified inclusion criteria.

**Table S2.**

**Title:** Demographic and Clinical Characteristics of those who returned a FIT Kit.

| **Characteristics** | **Overall, *n* (%)**  **total kits returned = 339** |
| --- | --- |
| **Sex** |  |
| Male | 124 (36.6) |
| Female | 215 (63.4) |
| **Age Range** |  |
| ≥25 | 7 (2.1) |
| 26-35 | 46 (13.6) |
| 36-45 | 68 (20.1) |
| 46-55 | 82 (24.2) |
| 56-65 | 65 (19.2) |
| 66-75 | 60 (17.7) |
| >75 | 8 (2.4) |
| **Anastomosis** |  |
| No | 227 (66.96) |
| Yes | 72 (21.24) |
| Unknown / Not Reported | 40 (11.80) |
| **GI Co-Morbidities** |  |
| Haemorrhoids/Piles | 7(2.06) |
| Irritable Bowel Disease (IBD) | 0 (0.00) |
| Diverticulosis | 12 (3.54) |
| Ulcerative Colitis | 2 (0.59) |
| Angiodysplasia | 0 (0.00) |
| None Reported | 138 (40.70) |
| Unknown / Unreported | 177 (52.21) |
| Other (Crohn’s Disease, Coeliac Disease, Muir-Torre Syndrome) | 1 (0.29) |

**Table S3.**

**Title:** Summary of findings from a two-sample t-test with unequal variances (*p*-value ≤0.05) to examine variance of age between those who returned a FIT kit and those who did not.

|  | Observations  (n) | Mean | Std. error  (%) | Std. dev. | | [95% CI]  Lower Upper | |
| --- | --- | --- | --- | --- | --- | --- | --- |
| Returned a FIT kit (x) | 339 | 52.27 | 0.76 | 13.99 | 49.78 | | 52.76 |
| Did not return a FIT kit (y) | 174 | 44.77 | 0.97 | 12.81 | 42.85 | | 46.69 |
| Combined | 513 | 49.07 | 0.62 | 13.93 | 47.86 | | 50.28 |
| diff |  | 6.5 | 1.23 |  | 4.08 | | 8.92 |
| diff = mean (x) - mean (y) | |  |  |  |  | | t = 5.2715 |
| H0: diff = 0.00 | |  | Satterthwaite’s degrees of freedom (df) = 377.283 | | | | |

CI: Confidence Interval

**References**

1. Waller J, McCaffery K, Forrest S, Szarewski A, Cadman L, Austin J, et al. Acceptability of unsupervised HPV self-sampling using written instructions. J Med Screen. 2006;13(4):208-13.
